# Piceatannol enhances polymyxin B efficacy against *Escherichia coli* O157:H7 via inhibition of biofilm formation

**DOI:** 10.3389/fmicb.2026.1933569

**Published:** 2026-10-16

**Authors:** Jing Xu, Yaxin Zhou, Wenkui Bao, Zhanqing Ma, Zixuan Shang, Hongxing Zhang, Weiwei Wang, Bing Li, Yubin Bai, Jiyu Zhang

**Affiliations:** 1 Key Laboratory of New Animal Drug Project of Gansu Province, Key Laboratory of Veterinary Pharmaceutical Development of the Ministry of Agriculture and Rural Affairs, New Veterinary Drugs Engineering Research Center of Gansu Province, Key Laboratory of New Veterinary Drugs Engineering of CAAS, Laboratory Animal Science Data Center of Gansu Province, Key Laboratory of Animal Genetics and Breeding on Tibetan Plateau, Ministry of Agriculture and Rural Affairs, Lanzhou Institute of Husbandry and Pharmaceutical Sciences of CAAS, Lanzhou, Gansu, China; 2 Qinghai Provincial Agricultural Product Quality and Safety Inspection Center, Xining, Qinghai, China; 3 College of Life Science and Food Engineering, Hebei University of Engineering, Handan, Hebei, China

**Keywords:** biofilm, EPS, *Escherichia coli* O157:H7, piceatannol, polymyxin B

## Abstract

**Introduction:**

*Escherichia coli* O157:H7 (*E. coli O157*) poses a severe global health threat, particularly due to its ability to form recalcitrant biofilms and the paradoxical exacerbation of life‑threatening haemolytic uraemic syndrome by conventional antibiotics. This study identifies piceatannol as an effective agent that suppresses *E. coli* O157 biofilm formation *in vitro* and acts as an adjuvant to enhance antimicrobial efficacy.

**Methods:**

The effects of piceatannol on biofilm formation, swarming motility, and exopolysaccharide (EPS) and protein production were evaluated *in vitro*. Pharmacological screening identified an effective combination with polymyxin B, which was validated in a murine infection model by assessing bacterial loads in visceral organs (liver and spleen), ileal villous morphology, and survival rates.

**Results:**

Piceatannol suppressed biofilm formation by inhibiting swarming motility and reducing EPS and protein production, thereby weakening biofilm integrity and enhancing antimicrobial efficacy. *In vivo*, the piceatannol–polymyxin B combination reduced bacterial loads in visceral organs, restored ileal villous morphology, and improved survival more effectively than monotherapy.

**Discussion:**

These findings establish piceatannol as an effective adjuvant that enhances polymyxin B efficacy against E. coli O157. By disrupting biofilm integrity and sensitizing bacteria to polymyxin B, the piceatannol–polymyxin B combination represents a promising strategy for treating E. coli O157 infections.

## 1 Introduction

Enterohemorrhagic *Escherichia coli* O157:H7 (*E. coli* O157) remains a globally dominant and highly virulent foodborne pathogen, responsible for millions of cases of severe haemorrhagic colitis and life-threatening haemolytic uremic syndrome (HUS) annually (Nemati et al., 2025). The public health burden of this pathogen is exacerbated by its exceptional ability to form resilient biofilms throughout the agrifood continuum, spanning bovine farm environments and food processing equipment to stainless-steel kitchen utensils and, most critically, the human intestinal mucosa (Vogeleer et al., 2014; Giaouris et al., 2014; Croxen and Finlay, 2010). This sessile lifestyle confers a formidable defence against conventional interventions, shielding the pathogen from host immune surveillance and creating reservoirs for the propagation of antimicrobial-resistant strains (Bridges et al., 2018). The management of *E. coli* O157 infections is severely limited by the fact that standard antibiotics are contraindicated. Therapeutic administration of bactericidal agents often triggers the inadvertent release of Shiga toxins, thereby exacerbating systemic complications and elevating the risk of HUS, while simultaneously exerting selective pressure that accelerates the emergence of resistance (Buchanan and Doyle, 1997; Jeon et al., 2025; Yang et al., 2025). Although the acquisition of resistance genes via horizontal gene transfer or chromosomal mutations represents the classic evolutionary pathway, biofilm-mediated antibiotic tolerance has emerged as an equally pivotal, yet mechanistically distinct, driver of treatment failure (Davies and Davies, 2010; Høiby et al., 2010; Brauner et al., 2016). Distinct from genetic resistance, biofilm tolerance results from physical, metabolic, and communicative barriers, including extracellular polymeric substance diffusion restriction, persister cell formation, and quorum sensing (QS)-regulated virulence, thereby nullifying conventional therapies even at susceptible MICs (Sutherland, 2001; Rutherford and Bassler, 2012; Flemming et al., 2023). Overcoming these therapeutic limitations therefore necessitates moving beyond conventional bactericidal strategies toward adjuvant-based approaches that restore antibiotic susceptibility and suppress the emergence of resistance (Zhang et al., 2025; Xu et al., 2026). In this context, pairing natural bioactive compounds with antibiotics offers a promising strategy to achieve synergistic bacterial clearance while circumventing toxin induction and curtailing resistance selection (Zhou et al., 2026).

Piceatannol, a natural hydroxylated stilbene predominantly found in passion fruit (*Passiflora edulis Sims*) seeds and grapes, has garnered increasing attention for its pleiotropic bioactivities, including antioxidant (Tang and Chan, 2014), anti-inflammatory (Takayuki et al., 2017), anticancer (Banik et al., 2020), and metabolic regulatory effects (Seyed et al., 2016). Despite these advances, the potential of piceatannol to combat bacterial infections, particularly its effect on bacterial virulence factors such as biofilm formation, remains largely unexplored. During our screening for natural compounds that disrupt bacterial pathogenesis, we interestingly discovered that piceatannol exhibits effective biofilm-inhibitory activity in *E. coli* O157. These results prompted us to investigate the molecular mechanisms underlying this phenotype, with a specific focus on its capacity to suppress biofilm matrix synthesis, attenuate motility, and act as an adjuvant to potentiate polymyxin B. Consequently, to address the clinical challenge of polymyxin-resistant *E. coli* O157 infections, we systematically screened piceatannol in combination with a panel of conventional antibiotics. Polymyxin B emerged as the most efficacious partner, exhibiting superior synergy with piceatannol to restore susceptibility against this pathogen. By employing a phenotype-driven screening platform targeting biofilm biomass and metabolic viability, we characterized the impact of this optimal combination on exopolysaccharide (EPS) and extracellular protein (ECP) production, motility and ultrastructural architecture. Crucially, the synergistic efficacy was validated in a murine *E. coli* O157 infection model. Collectively, our findings reveal a distinct strategy in which piceatannol potentiates polymyxin B activity against early-stage biofilm-associated colonization, providing a potential preventive avenue to reduce selective pressure associated with antimicrobial resistance.

## 2 Materials and methods

### 2.1 Materials and bacteria

The piceatannol used in this study was from MedChemExpress. Polymyxin B (≥98%), tetracycline hydrochloride (≥98%), ciprofloxacin (≥98%), gentamicin sulfate (≥98%), aztreonam (≥98%), ceftriaxone sodium (≥98%), cefotaxime (≥98%), and doxycycline hydrochloride (≥98%) to be tested and used in this study were purchased from Shanghai Yuanye Biotechnology Co., Ltd. (Shanghai, China). All antibiotics were of analytical grade or higher and used as received. Piceatannol was dissolved in dimethyl sulfoxide (DMSO) to yield a 40 mg/mL stock solution. The final DMSO concentration in the LB medium was maintained below 1%. For control experiments, an equal volume of DMSO without the compound was added to account for the solvent effect.

*E. coli* O157:H7 (ATCC 43895; stx1 + stx2+), was obtained from the American Type Culture Collection (ATCC). All *in vitro* and *in vivo* experiments were conducted exclusively using this single strain. The growth medium consisted of AB medium (0.05 M MgSO₄, 0.3 M NaCl, and 0.2% vitamin-free Casamino Acids per litre) supplemented with 1 mM L-arginine, 1% phosphate buffer (pH 7.2), and 1% glycerol.

### 2.2 Minimum inhibitory concentration (MIC)

The two-fold serial broth microdilution method was used to determine the minimum inhibitory concentration (MIC) of piceatannol against *E. coli* O157 in a 96-well plate. Briefly, Mueller-Hinton broth containing 0.15% (w/v) agar (MHBA) was prepared, and piceatannol concentrations ranged from 256 μM to 8 μM. Overnight *E. coli* O157 cultures were diluted to approximately 1 × 10^6^ CFU/mL in MHBA. Each well of the 96-well plate contained 100 μL of MHBA and 100 μL of the bacterial suspension (1 × 10^6^ CFU/mL), resulting in a final inoculum of approximately 5 × 10^5^ CFU/mL per well. Positive control wells contained *E. coli* O157 (5 × 10^5^ CFU/mL) in MHBA without piceatannol. Wells containing 200 μL of uninoculated MHBA served as blanks. The plates were incubated statically at 37 °C for 24 h. The MIC was defined as the lowest concentration of piceatannol at which no visible bacterial growth (i.e., no change in turbidity) was observed. All experiments were performed in sextuplicate.

### 2.3 Biofilm inhibition

Biofilm inhibition assays with piceatannol were assessed using the crystal violet (CV) staining assay (O'toole, 2011). *E. coli* O157 was initially cultured at 37 °C for 24 h. Following this, the bacterial suspension was standardized to an OD600 of 0.01 and dispensed into white 96-well plates (Corning Costar® 3,599, Corning, Kennebunk, Maine, USA) containing piceatannol at concentrations ranging from 16 to 128 μM. After a subsequent 24-h incubation at 37 °C, non-adherent cells were removed by three washes with PBS (pH 7.2). The remaining biofilms were fixed with methanol, stained with 0.1% (w/v) CV for 30 min, and destained with tap water. Bound dye was solubilized in 150 μL of 95% ethanol, and absorbance was measured at 595 nm. All assays were performed in triplicate.

### 2.4 Biofilm eradication

*E. coli* O157 was initially cultured statically at 37 °C for 24 h. The resulting bacterial suspension was then adjusted to an OD600 of 0.01, and 150 μL aliquots were transferred to 96-well microplates (Corning Costar® 3,599, Corning Inc., USA). Following a 24-h incubation at 37 °C to permit biofilm formation, the wells were gently washed three times with PBS (pH 7.2). Pre-formed biofilms were subsequently exposed to piceatannol at concentrations ranging from 16 μM to 128 μM and incubated for an additional 24 h. After treatment, the medium was aspirated, and the biofilms were washed three times with sterile PBS. The adherent cells were fixed with methanol for 30 min and stained with 0.1% (m/v) crystal violet (CV) for 10 min. Unbound dye was removed by three PBS washes, and the remaining CV was solubilized in 150 μL of 95% ethanol. Absorbance was measured at 595 nm using a microplate reader. All assays were performed in triplicate.

### 2.5 Bacterial growth curve analysis

The effect of piceatannol on the growth curve of *E. coli* O157 was monitored using a microplate reader. Briefly, overnight cultures were diluted 1:100 into fresh LB medium containing piceatannol at final concentrations ranging from 32 to 128 μM. The cultures were then dispensed into 96-well flat-bottom plates and incubated at 37 °C with continuous shaking. The optical density (OD₆₀₀) was measured automatically every 2 h over a 12 h period. Wells containing uninoculated medium served as blanks for background correction, while bacterial cultures without piceatannol served as control. To further assess bacterial viability, CFU assays were performed in parallel. At designated time points (0, 2, 4, 6, 8, 10, and 12 h), aliquots were collected from each treatment group, serially diluted in sterile PBS, and plated onto LB agar. Colonies were counted after incubation at 37 °C for 24 h. All experiments were performed in triplicate.

### 2.6 Shiga toxin production

Shiga toxin production was measured using a Shiga toxin ELISA kit (YS-X42907; Yushao Biology Co., Ltd.) according to the manufacturer’s instructions. Overnight cultures of *E. coli* O157 were adjusted to an initial OD600 of approximately 0.2 using a cell density meter (WPA CO8000, Biochrom Ltd., Cambridge, UK). Thereafter, 3 mL aliquots of the culture were distributed into separate tubes and treated as follows: (i) blank control (1% DMSO); (ii) polymyxin B (0.5 μg/mL; dissolved in sterile water); (iii) piceatannol (128 μM; dissolved in DMSO, final DMSO concentration 1%); (iv) polymyxin B (0.5 μg/mL) combined with piceatannol (128 μM; final DMSO 1%). The cultures were incubated at 37 °C for 12 h. After incubation, cells were harvested by centrifugation at 6000 × *g* for 10 min, and 200 μL of the supernatant was filtered through a 0.22 μm filter for Shiga toxin detection. All experiments were performed in triplicate.

### 2.7 Metabolic activity

Metabolic activity of *E. coli* O157 was assessed using the Alamar Blue assay (Xu et al., 2026) following exposure to piceatannol concentrations ranging from 32 to 128 μM. Briefly, cells were harvested from 48-well plates into fresh microcentrifuge tubes, centrifuged, washed with PBS, and resuspended in 1 mL of PBS. Aliquots of the cell suspension were then transferred to a 96-well plate, followed by the addition of 10 μL of Alamar Blue dye. After incubation at 37 °C in the dark for 1 h, absorbance was measured at 570 nm and 600 nm using a microplate reader. A blank control consisting of PBS with dye only was included to calculate metabolic activity based on the following formula:

$$\text{Metabolic activity}\;(\%)=\frac{\left(\begin{array}{l} \left(E_{\mathrm{oxi}(\mathrm{OD}600)}\times T_{\mathrm{OD}570}\right)\\ -(E_{\mathrm{oxi}(\mathrm{OD}570)}\times T_{\mathrm{OD}600}) \end{array}\right)}{\left(\begin{array}{l} \left(E_{\mathrm{red}(\mathrm{OD}570)}\times B_{\mathrm{OD}600}\right)\\ -(E_{\mathrm{red}(\mathrm{OD}600)}\times B_{\mathrm{OD}570}) \end{array}\right)}\times 100\%$$

Eoxi_(OD570)_ — extinction coefficient of AB in its oxidized form at 570 nm = 80,586;

Ered_(OD570)_ — extinction coefficient of AB in its reduced form at 570 nm = 155,677;

Eoxi_(OD600)_ — extinction coefficient of AB in its oxidized form at 600 nm = 117,216;

Ered_(OD600)_ — extinction coefficient of AB in its reduced form at 570 nm = 14,652;

B — blank; T — samples.

### 2.8 Scanning electron microscopy (SEM) analysis

Biofilm morphology was examined via scanning electron microscopy (SEM) with slight modifications to established protocols (Bai et al., 2025). Concisely, bacterial suspensions were inoculated into 96-well plates containing cell culture slides and exposed to 32–128 μM piceatannol. Following a 24-h incubation at 37 °C to facilitate biofilm formation, the slides were gently washed three times with sterile PBS to dislodge planktonic cells and loosely attached debris. The slides were then carefully removed, subjected to sequential fixation and graded ethanol dehydration, and sputter-coated with gold. Finally, samples were imaged using an FEI Versa 3D SEM (Thermo Fisher Scientific, USA).

### 2.9 Assessment of biofilm viability via fluorescence staining

Following an established protocol (Duarte et al., 2024), *E. coli* O157 suspensions (adjusted to an OD₆₀₀ of 0.01) were inoculated into 6-well plates and treated with varying concentrations of piceatannol (32–128 μM). Following static incubation at 37 °C for 24 h, the supernatants were removed, and the wells were gently washed twice with PBS (pH 7.2). The biofilms were subsequently stained using the BacLight Live/Dead viability kit (L7012, Invitrogen™, Thermo Fisher Scientific, USA) according to the instructions of the manufacturer. Briefly, SYTO9 and propidium iodide (PI) stock solutions were diluted in PBS, and 500 μL of the dye mixture was added to each well. After a 15 min incubation in the dark, the staining solution was removed, and the wells were left to dry completely. Finally, the biofilms were visualized under a confocal laser scanning microscope (CLSM; Zeiss LSM800, Carl Zeiss AG, Tokyo, Japan) using excitation/emission wavelengths of 480/500 nm for SYTO9 and 490/635 nm for PI. Random fields of view were selected for image acquisition.

### 2.10 Bacterial motility

The impact of piceatannol on *E. coli* O157 motility was assessed using a semi-solid agar assay (Lee et al., 2014). Briefly, an overnight culture was standardized to an OD₆₀₀ of 1.0. Subsequently, 1 μL of the bacterial suspension was spotted onto 0.3% semi-solid LB agar plates supplemented with varying concentrations of piceatannol (32–128 μM). Following incubation at 37 °C for 24 h, motility was quantified by measuring the migration halo diameter relative to the control group. Each experiment was performed in triplicate.

### 2.11 EPS production

The quantification of EPS was performed using a ruthenium red staining assay according to the previous method (Adnan et al., 2020). Briefly, *E. coli* O157 cultures exposed to varying concentrations of piceatannol were seeded into 96-well plates and incubated at 37 °C for 24 h to allow biofilm formation. Following three washes with PBS, 200 μL of 0.01% (w/v) ruthenium red solution was added to each well (with blank wells receiving staining solution only). After a 60 min dark incubation at 37 °C, the unbound dye was transferred to a fresh plate, and the absorbance was read at 450 nm. Three independent biological replicates were conducted for each treatment. The EPS inhibition rate was subsequently calculated based on the following formula:

$$\mathrm{EPS}\;\text{Inhibition}\;(\%)=\frac{\left(\mathrm{AS}-\mathrm{AP}\right)}{\left(\mathrm{AB}-\mathrm{AP}\right)}\times 100\%$$

AB: Absorbance of the blank control solution;

AS: Absorbance of the sample solution;

AP: Absorbance of the positive control (untreated *E. coli*) solution.

### 2.12 ECP production

The extracellular protein (ECP) content of biofilms was quantified using the Bradford assay, following a previously established protocol (Siddique et al., 2020). A protein standard was prepared at a stock concentration of 0.2 mg/mL. To construct the standard curve, 0–20 μL of the standard (corresponding to 0, 2, 4, 6, 8, 12, 16, and 20 μL) was dispensed into a 96-well plate, and each well was adjusted to a final volume of 20 μL with PBS. Simultaneously, *E. coli* O157 suspensions treated with piceatannol at concentrations ranging from 16 to 256 μM were incubated statically in 96-well plates at 37 °C for 24 h. Following cultivation, the samples were appropriately diluted, and 20 μL of each diluted sample was transferred to the assay plate. Subsequently, 200 μL of G250 dye reagent was added to each well, and the plates were allowed to stand at room temperature for 5 min. Absorbance at 595 nm was measured using a microplate reader, and the ECP concentration was calculated by interpolating from the standard curve.

### 2.13 Combination antibacterial activity

The minimum inhibitory concentrations (MICs) of various antibiotics against *E. coli O157* were determined in accordance with the Clinical and Laboratory Standards Institute (CLSI) document M07, “Methods for Dilution Antimicrobial Susceptibility Tests for Bacteria That Grow Aerobically”. The MIC values were as follows: Cefotaxime (0.125 μg/mL), tetracycline hydrochloride (0.5 μg/mL), ciprofloxacin (0.015 μg/mL), gentamicin sulfate (8 μg/mL), aztreonam (0.125 μg/mL), ceftriaxone sodium (0.0625 μg/mL), and doxycycline hydrochloride (1 μg/mL). The MIC of polymyxin B was found to be 1 μg/mL. The synergistic antibacterial potential of piceatannol was evaluated in combination with conventional antibiotics following the method described by Bai et al. (2022). Briefly, *E. coli* O157 was cultured overnight and subsequently diluted to an OD₆₀₀ of 0.01. These bacterial suspensions were then exposed to sub-inhibitory concentrations of antibiotics (1/2, 1/4, and 1/8 MIC) either in the absence or presence of piceatannol. Following incubation at 37 °C for 16–18 h, the metabolic activity of the mixed cultures was quantified using the Alamar Blue assay.

### 2.14 *In vivo* synergy of antibiotic combinations

Female 6-week-old C57BL/6 J mice (18–22 g) were procured from the Lanzhou Veterinary Research Institute, Chinese Academy of Agricultural Sciences. All animal procedures were conducted in strict accordance with the guidelines for the care and use of laboratory animals and were approved by the Institutional Animal Care and Use Committee of the Lanzhou Institute of Husbandry and Pharmaceutical Sciences of CAAS (Approval No. 2025-25). Following a 3-day acclimatization period, mice were randomized into four groups (*n* = 12 per group): Control, Polymyxin B, Piceatannol, and a combination group (Piceatannol + Polymyxin B). *E. coli* O157 was cultured overnight and sub-cultured once before use. The bacterial suspension was adjusted to a 0.5 McFarland standard (equivalent to 1.0 × 10^8^ CFU/mL) and further concentrated to a final density of 1.5 × 10^9^ CFU/mL. On day 1, mice were intraperitoneally challenged with 0.2 mL/10 g of the bacterial suspension. One hour post-infection, polymyxin B (1.5 mg/mL) was administered intraperitoneally alone or in combination with piceatannol (20 mg/kg) to the respective groups. Survival was recorded daily over a 7-day period, and Kaplan–Meier survival curves were generated. On day 7, the remaining mice were euthanised via cervical dislocation under isoflurane anesthesia (RWD, China). Target tissues were harvested and preserved in appropriate fixatives or frozen for subsequent analysis, including bacterial load quantification (plate counting method).

Spleen, liver, and ileum tissues were fixed in 4% paraformaldehyde (Servicebio, China), dehydrated in a graded ethanol series, and cleared in xylene. The tissues were subsequently embedded in molten paraffin, sectioned into 5–8 μm slices using a rotary microtome, and stained with haematoxylin and eosin (H&E) for histopathological evaluation (Guo et al., 2023).

### 2.15 Statistical analysis

All statistical evaluations and graphical representations were generated using GraphPad Prism (version 10.0, GraphPad Software, San Diego, CA, USA). Data normality and homogeneity of variances were assessed prior to analysis. Data are presented as mean ± standard deviation (SD) of *n* independent biological replicates. For experiments involving multiple groups, statistical significance was assessed using either a Kruskal–Wallis test (for non-parametric data) followed by Dunn’s *post hoc* test or a two-way ANOVA with Tukey’s *post hoc* test (for parametric data) for multiple comparisons. For comparisons between two groups, an unpaired t-test was used. For survival analysis, the Log-rank (Mantel-Cox) test was used. Exact *p* values are provided in the figures, tables, and text. Statistical significance was defined as *p* < 0.05.

## 3 Results

### 3.1 Piceatannol inhibits *E. coli* O157 biofilm formation

As shown in Figure 1A, piceatannol treatment resulted in a significant, concentration-dependent inhibition of *E. coli* O157 biofilm formation. The biofilm inhibition rate increased progressively with rising concentrations (ranging from 16 μM to 128 μM). Notably, treatment with 128 μM piceatannol reduced biofilm formation by approximately 98%. However, as shown in Figure 1B, piceatannol exhibited no significant disruptive effect against pre-formed mature biofilms. The biofilm retention rate remained consistently high (nearly 100%) across all tested concentrations (16 μM to 128 μM), indicating that piceatannol fails to disrupt established mature biofilms within this concentration range. Fluorescent microscopic analysis using the Live/Dead assay (Figure 1C) complemented these observations. While the untreated control group exhibited dense green fluorescence indicative of metabolically active, live cells, piceatannol treatment caused a marked shift in fluorescence signals. Specifically, treatment with 64 μM and 128 μM piceatannol led to a substantial increase in red fluorescence (dead cells) and a corresponding reduction in green fluorescence (live cells), demonstrating that piceatannol impairs *E. coli* O157 surface colonization and subsequently attenuates biofilm development in a dose-dependent manner. Finally, morphological changes were observed via scanning electron microscopy (SEM) (Figure 1D). Untreated biofilms displayed intact structures with tightly packed cell clusters. In contrast, treatment with piceatannol (32–128 μM) compromised the extracellular matrix integrity, correlating with altered biofilm architecture and increased intercellular spacing.

**Figure 1:**
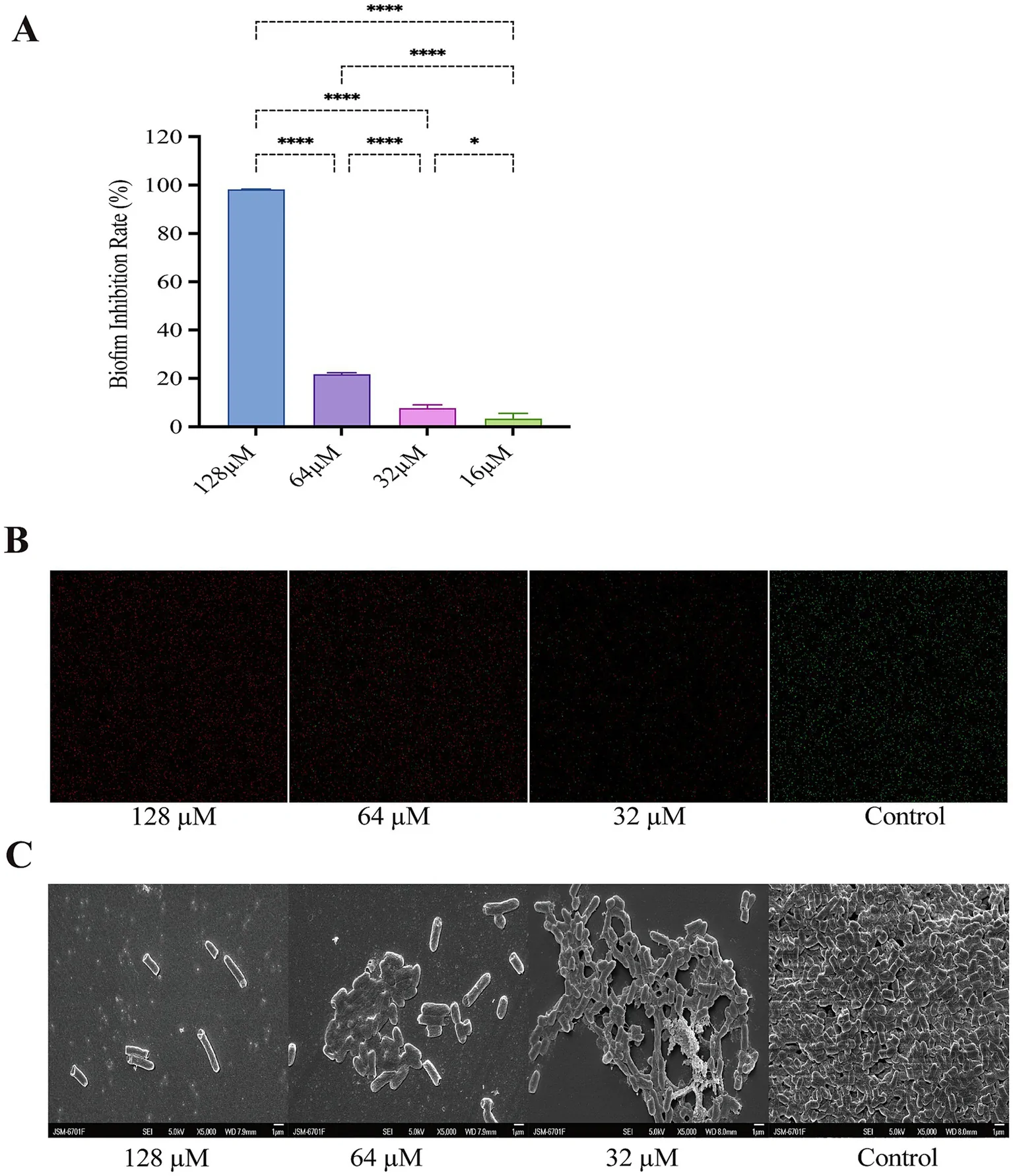
Effects of piceatannol on *E. coli* O157 biofilm formation. **(A)** Biofilm inhibition rate of *E. coli* O157 after treatment with increasing concentrations of piceatannol. **(B)** Biofilm retention rate of *E. coli* O157 after treatment with increasing concentrations of piceatannol. (*n* = 3 per group; Data represent means ± SD; *p < 0.05, **p < 0.01, ***p < 0.001, ****p < 0.0001); **(C)** Fluorescent microscopic images showing live/dead biofilm cells after treatment with different concentrations of piceatannol (Green indicates live cells, Red indicates dead cells). **(D)** Scanning electron microscopy (SEM) images of biofilms treated with different concentrations of piceatannol (Scale bar = 1 μm).

### 3.2 Growth and motility suppression

The MIC of piceatannol against *E. coli* O157 was determined to be >256 μM, confirming its lack of direct bactericidal activity. Given this high MIC, the sub-MIC effects on bacterial proliferation were further examined. As shown in Figure 2A, the growth dynamics of *E. coli* O157 in response to piceatannol revealed that while the untreated control exhibited normal growth kinetics with an optical density (OD₆₀₀) plateauing at approximately 12 h, piceatannol treatment significantly impeded bacterial proliferation in a concentration-dependent manner. The highest concentration tested (128 μM) exerted the most significant suppressive effect, maintaining OD₆₀₀ values below those of the control throughout the assay. Corroborating these growth profiles, parallel CFU assays (Figure 2B) demonstrated a time-dependent reduction in bacterial viability. Although initial bacterial loads (0–4 h) were comparable across all groups, the 128 μM treatment group exhibited a significantly lower Log₁₀ CFU/mL count compared to the untreated control at later time points (specifically at 12 h), confirming the growth-suppressive effects of piceatannol at sub-MIC concentrations. Corroborating these growth profiles, parallel measurements in Figure 2C reveal a marked reduction in total metabolic signal. Relative to the robust metabolic activity observed in the untreated control group, exposure to escalating doses of piceatannol resulted in a progressive and dose-dependent decline in metabolic output, as measured by the Alamar Blue assay. Specifically, metabolic activity was reduced to 31.3% at 32 μM, 24.3% at 64 μM, and 11.7% at 128 μM (adjusted *p* < 0.0001 vs. control). Beyond planktonic growth, piceatannol also attenuated the motile behavior of *E. coli* O157. Figures 2D,E depict the impact of piceatannol on swarming motility. On semi-solid agar, control bacteria formed expansive, diffuse swarms radiating from the inoculation site. Piceatannol treatment restricted this outward expansion in a dose-dependent manner. The 128 μM group formed the smallest swarm diameter, indicating that piceatannol suppresses swarming motility phenotype on semi-solid surfaces.

**Figure 2:**
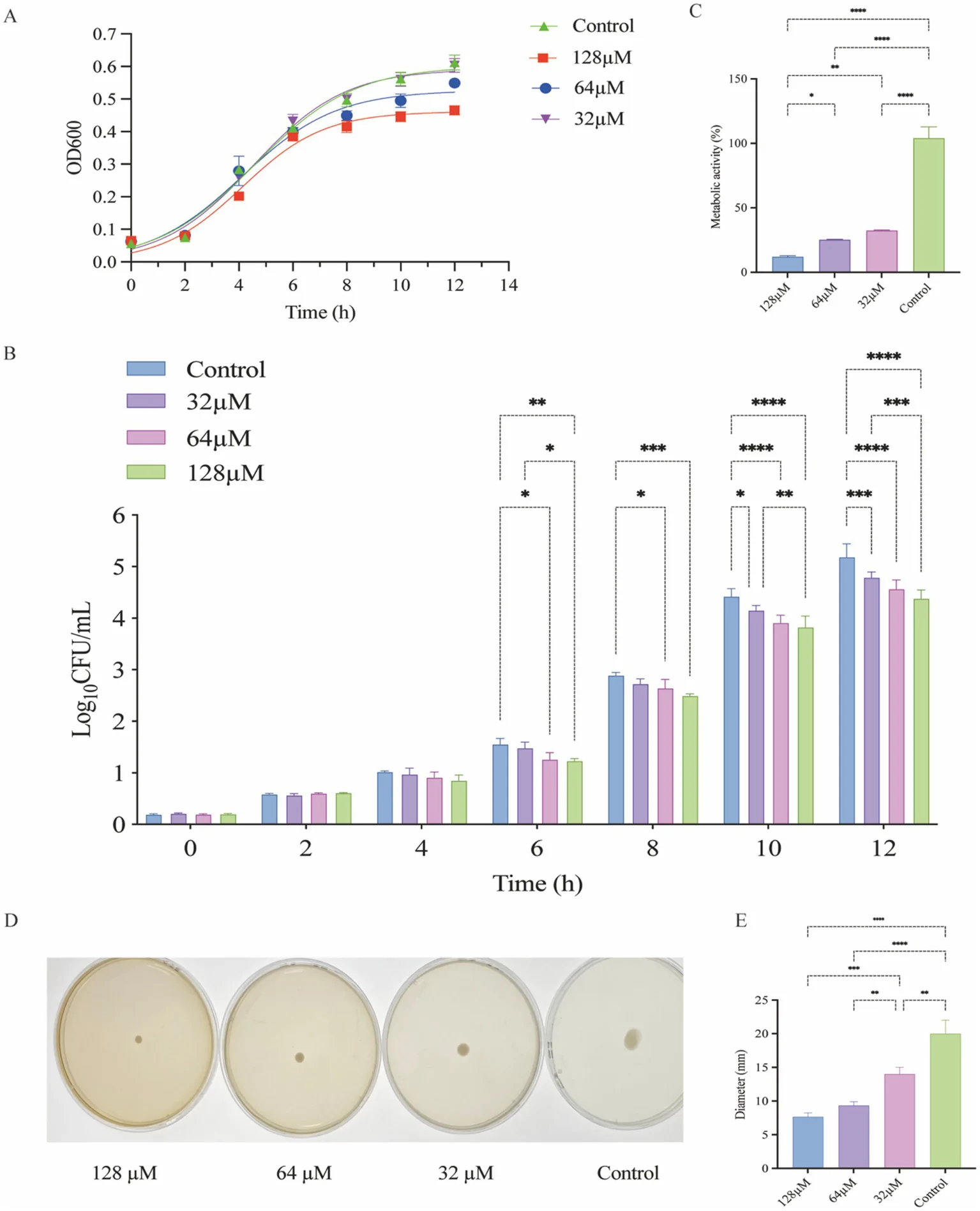
Piceatannol inhibits the growth and viability of *E. coli* O157. **(A)** Growth curves of *E. coli* O157 treated with different concentrations of piceatannol (32 μM, 64 μM, and 128 μM) or untreated (Control). **(B)** CFU counts of *E. coli* O157 treated with different concentrations of piceatannol (32 μM, 64 μM, and 128 μM). **(C)** Metabolic activity of *E. coli* O157 after treatment with piceatannol. **(D)** Representative images of swarming motility assays, showing the inhibitory effect of piceatannol on bacterial swarm expansion. **(E)** Quantification of swarm diameters, indicating a dose-dependent inhibition of *E. coli* O157 swarming motility by piceatannol (*n* = 3 per group; Data represent means ± SD; *p < 0.05, **p < 0.01, ***p < 0.001, ****p < 0.0001).

### 3.3 Piceatannol suppresses EPS and ECP production

Figure 3A illustrates the inhibitory effect of piceatannol on EPS production by *E. coli* O157. Treatment with increasing concentrations of piceatannol resulted in a dose-dependent reduction in EPS levels. Both 256 μM and 128 μM treatments effectively suppressed EPS synthesis, achieving inhibition rates of approximately 75%. However, further reductions in the compound concentration led to a marked attenuation of this inhibitory activity. Notably, the 16 μM treatment group exhibited a relatively modest inhibitory effect, with an inhibition rate of approximately 20%. Similarly, Figure 3B depicts the impact of piceatannol on ECP release. Piceatannol administration reduced this release in a concentration-dependent manner. Maximal inhibition was observed at 256 μM and 128 μM, where ECP release was reduced by roughly 60%. As the concentration decreased to 64 μM and 32 μM, the inhibitory effect diminished, with the 16 μM treatment group exhibiting a relatively modest inhibitory effect of approximately 20%.

**Figure 3:**
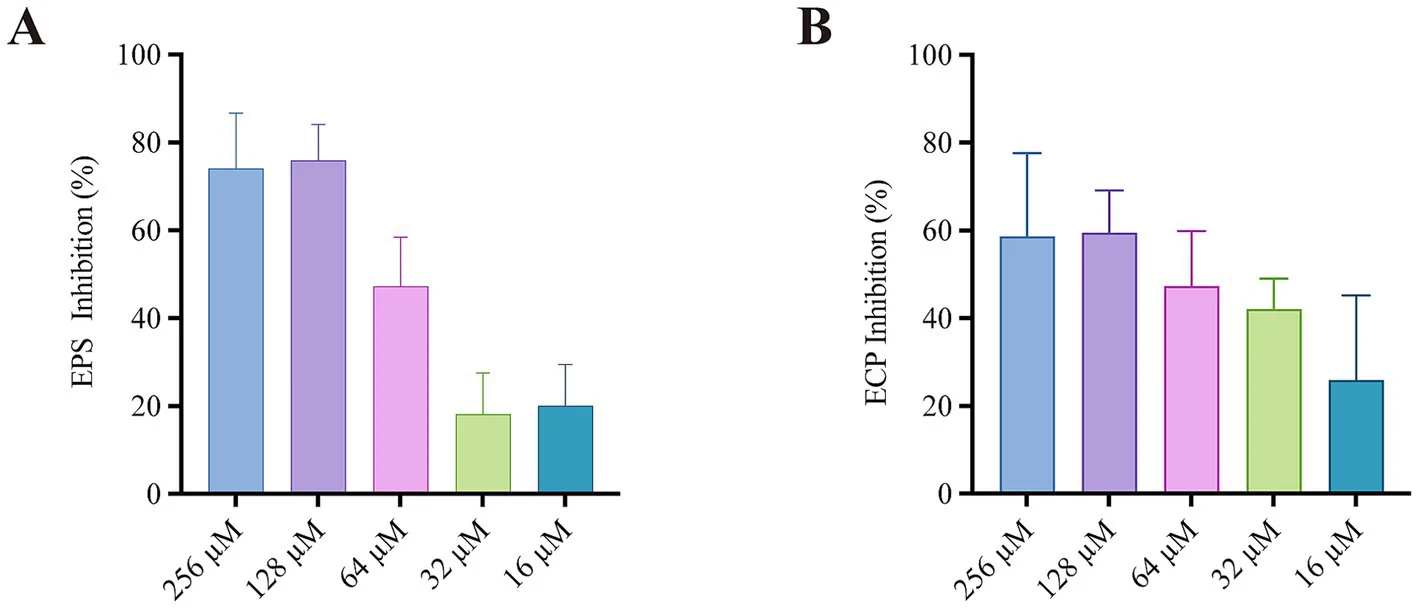
Piceatannol inhibits exopolysaccharide (EPS) production and extracellular protein (ECP) release in *E. coli* O157. **(A)** EPS inhibition rate of *E. coli* O157 after treatment with increasing concentrations of piceatannol. **(B)** ECP inhibition rate of *E. coli* O157 after treatment with increasing concentrations of piceatannol (*n* = 3 per group; Data represent means ± SD).

### 3.4 Combination effects of piceatannol and antibiotics

As shown in Figure 4, the effects of the antibiotic-piceatannol combination on the viability of *E. coli* O157 were evaluated. The antibiotics were tested at sub-inhibitory concentrations (1/2, 1/4, and 1/8 MIC) to determine whether piceatannol could enhance their antibacterial activity. In the majority of cases, the addition of piceatannol did not significantly impair bacterial viability compared to the antibiotics alone. Figure 4A illustrates a pronounced combined activity between piceatannol and polymyxin B. As expected, reducing the concentration of polymyxin B alone (blue bars) from 1/2 to 1/8 MIC resulted in a substantial increase in bacterial viability. However, the addition of piceatannol (red bars) effectively reversed this tolerance. Notably, at 1/2 and 1/4 MIC, piceatannol reduced bacterial viability from approximately 60% down to nearly 2 and 20%, respectively, enhanced growth-suppressive activity. Consistent with this potentiating effect on antibacterial activity, the combination of piceatannol and polymyxin B also markedly suppressed the production of Shiga toxin (Figure 5). Compared to the untreated control, treatment with piceatannol (128 μM) or polymyxin B (0.5 μg/mL) alone significantly reduced toxin levels. However, their combination exhibited the most potent inhibitory effect, decreasing toxin production to approximately 2 pg./mL (*p* < 0.01 compared to piceatannol alone; *p* < 0.001 compared to control). Similarly, Figure 4B demonstrates that piceatannol enhances the antibacterial activity of tetracycline hydrochloride. At sub-inhibitory concentrations (1/2 MIC), the combination treatment significantly lowered bacterial survival compared to the antibiotic monotherapy. Although the difference is eliminated at the 1/8 and 1/4 MIC, the overall trend supports the enhancing potential of piceatannol against *E. coli* O157 when combined with these antibiotics. However, for ciprofloxacin (Figure 4C), gentamicin sulfate (Figure 4D), aztreonam (Figure 4E), and ceftriaxone sodium (Figure 4F), the red bars (combination) showed bacterial viability profiles largely comparable to the blue bars (antibiotics alone), suggesting no substantial interference from piceatannol. Notably, the most striking antagonism was observed with cefotaxime (Figure 4G) and doxycycline hydrochloride (Figure 4H), where the combination with piceatannol led to a marked increase in bacterial survival, particularly at sub-inhibitory concentrations (1/2 and 1/4 MIC). For cefotaxime, the viability (antibiotic alone) rose from ~5% (1/2 MIC) and 25% (1/4 MIC) to ~12% and ~50%, respectively. And for doxycycline hydrochloride, the viability (antibiotic alone) rose from ~40% (1/2 MIC) and 60% (1/4 MIC) to ~80% and ~90%, respectively. Collectively, these results demonstrate that while piceatannol is compatible with some antibiotics, it can significantly antagonize the activity of others, particularly cefotaxime and doxycycline, at sub-inhibitory concentrations.

**Figure 4:**
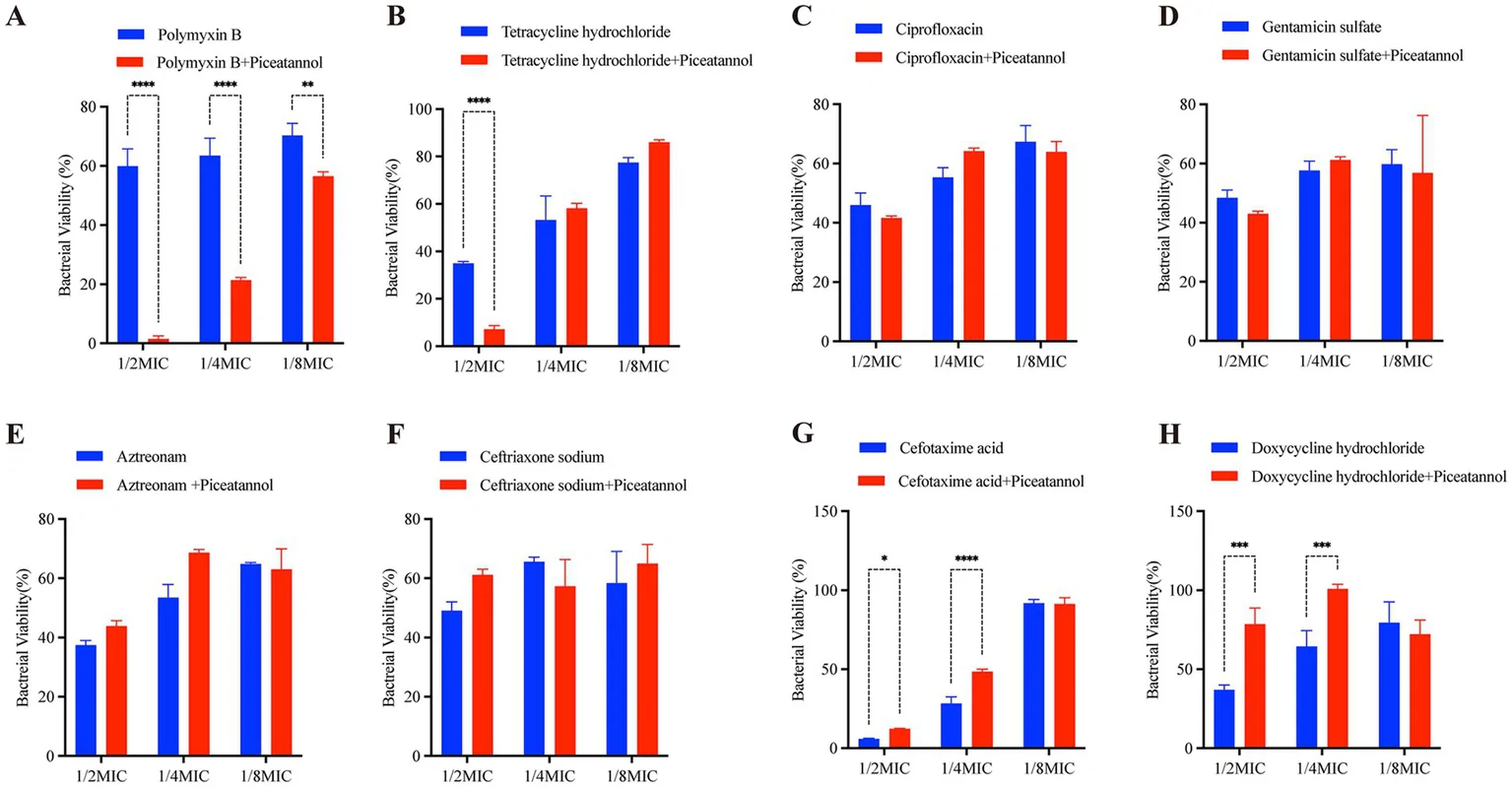
Effects of antibiotic-piceatannol combination on bacterial viability. Bar graphs **(A–H)** show the viability of *E. coli* O157 treated with eight common antibiotics (polymyxin B, tetracycline hydrochloride, ciprofloxacin, gentamicin sulfate, aztreonam, ceftriaxone sodium, cefotaxime, and doxycycline hydrochloride) at sub-inhibitory concentrations (1/2, 1/4, and 1/8 MIC), either alone or in combination with a fixed concentration of piceatannol (*n* = 3 per group; Data represent means ± SD; *p < 0.05, **p < 0.01, ***p < 0.001, ****p < 0.0001).

**Figure 5:**
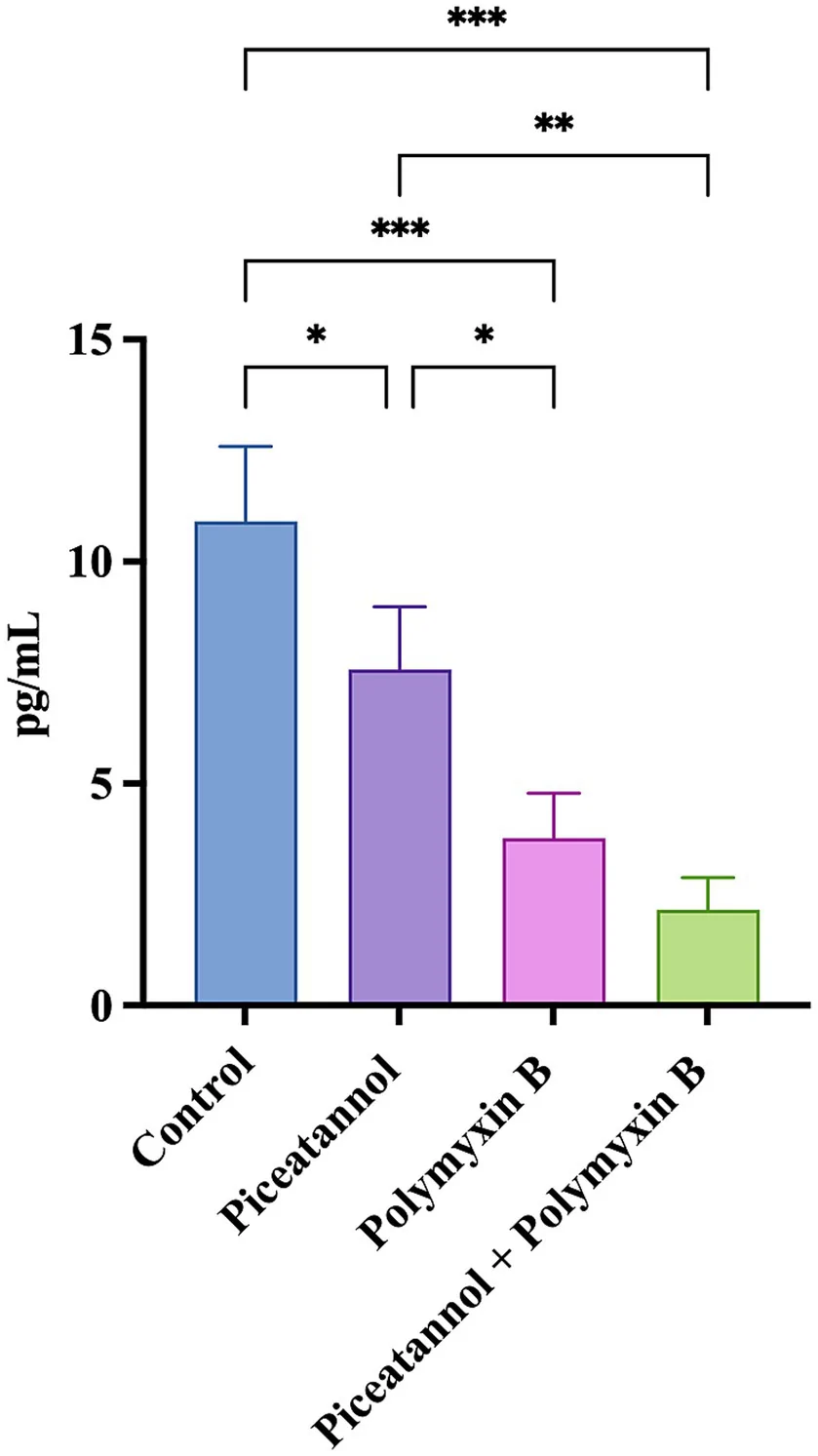
Effect of piceatannol and polymyxin B on Shiga toxin production by *E. coli* O157. Bacterial cultures were treated with piceatannol (128 μM), polymyxin B (0.5 μg/mL), or their combination. Data represent means ± SD (*n* = 3). *p < 0.05, **p < 0.01, ***p < 0.001.

### 3.5 *In vivo* efficacy of piceatannol-polymyxin B combination

Building on the *in vitro* combinatorial screening results (Figure 4), piceatannol demonstrated a pronounced synergistic interaction with polymyxin B, effectively reducing viability to nearly 2% at 1/2 MIC. Given this enhanced antibacterial effect and no observed antagonism *in vitro*, the piceatannol-polymyxin B combination was selected for further evaluation in a murine model of *E. coli* O157 infection. Figure 6 presents the *in vivo* protective efficacy of the piceatannol-polymyxin B combination in a murine model of *E. coli* O157 infection. The Kaplan–Meier survival curve (Figure 6A) demonstrated that while treatment with polymyxin B alone provided a significant survival benefit compared to the untreated model group, the combination therapy resulted in a further marked increase in protection, with the survival rate reaching 80% by day 7. Consistently, bacterial load quantification revealed that the combination treatment significantly reduced *E. coli* O157 colonization in both the liver (Figure 6B) and spleen (Figure 6C) compared to the model group and piceatannol monotherapy. Specifically, in the liver, the combination treatment achieved a significantly greater reduction in CFU compared to polymyxin B alone (*p* < 0.01), while in the spleen, the combination group exhibited a significantly lower bacterial count than both the model and piceatannol-only groups (*p* < 0.0001).

**Figure 6:**
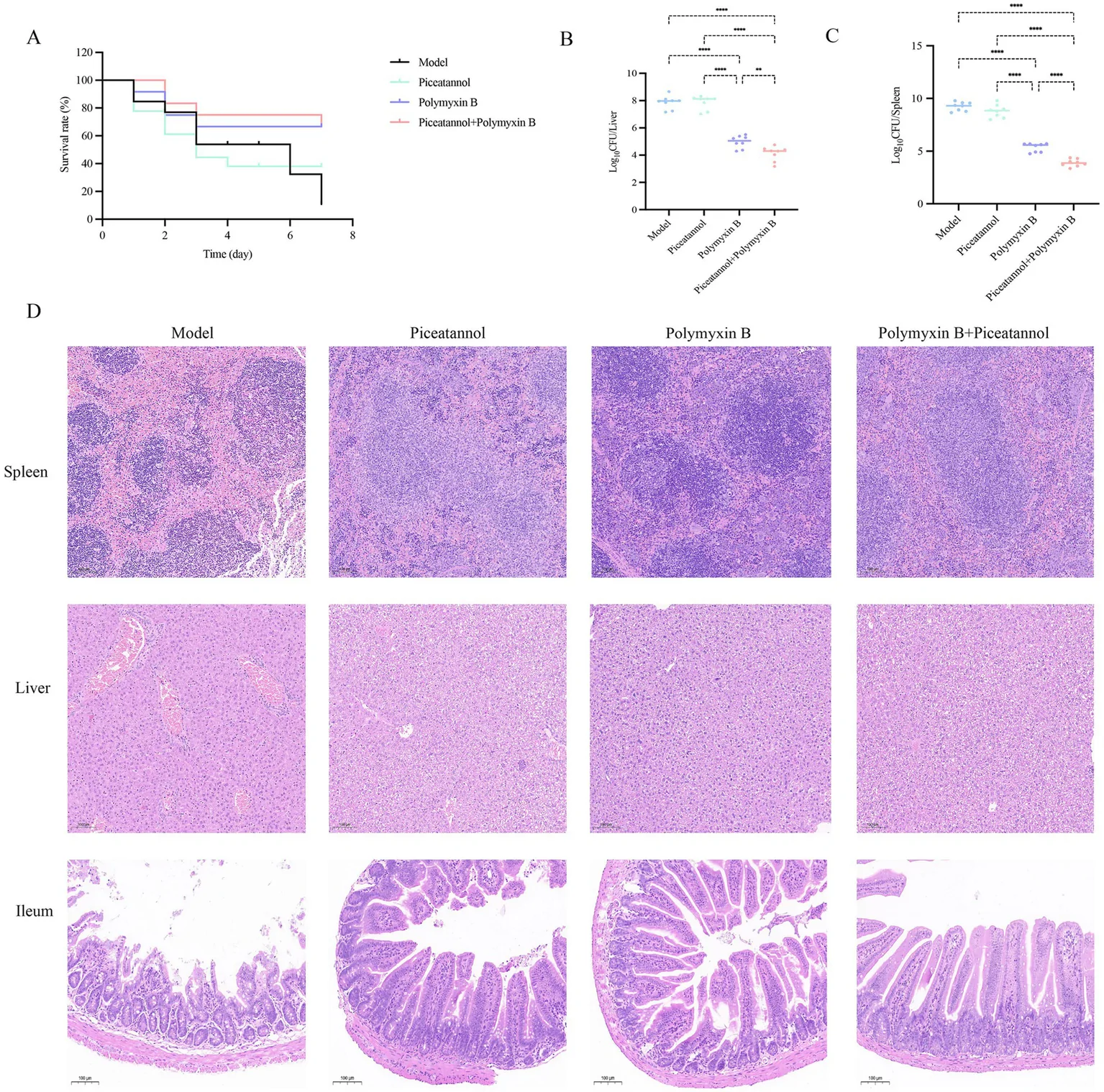
Efficacy of the piceatannol–polymyxin B combination against *E. coli* O157 infection in a murine model. **(A)** Survival rate of mice following *E. coli* O157 infection under different treatments. Mice (*n* = 12 per group) were treated with vehicle (Model), piceatannol alone, polymyxin B alone, or their combination. Statistical significance was assessed using the Log-rank (Mantel-Cox) test. **(B)** Bacterial loads in the liver of *E. coli* O157-infected mice after different treatments. Mice were treated with vehicle (Model), piceatannol alone, polymyxin B alone, or their combination. **(C)** Bacterial loads in the spleen of *E. coli* O157-infected mice after different treatments (*n* = 3 per group; Data represent means ± SD; *p < 0.05, **p < 0.01, ***p < 0.001, ****p < 0.0001). **(D)** Hematoxylin and eosin (H&E) staining of spleen, liver, and ileum from *E. coli* O157-infected mice with different treatments (Scale bar = 100 μm).

Furthermore, hematoxylin and eosin (H&E) staining (Figure 6D) revealed severe pathological damage in the model group across the spleen, liver, and ileum, characterized by extensive inflammatory cell infiltration, architectural disorganization, and focal necrosis. In the spleen, extensive and poorly demarcated hemorrhagic necrotic foci were observed alongside severe disruption of the normal splenic corpuscle architecture, indicating substantial infection-induced injury. In the liver, the model group exhibited disordered hepatic cord architecture with extensive inflammatory cell infiltration and punctate necrosis around central veins and portal areas. In the ileum, significant damage to the small intestinal mucosal structure was observed, manifested specifically as villous atrophy and disruption of the tissue structure. Treatment with piceatannol alone moderately reduced inflammatory cell infiltration and hepatocyte damage, showing a better outcome than the model, but it did not significantly alter the bacterial burden or substantially restore tissue architecture. In contrast, polymyxin B monotherapy markedly diminished haemorrhagic necrosis and considerably restored splenic structure, while also effectively suppressing hepatic inflammation and promoting partial recovery of intestinal villi. Most notably, the combination of piceatannol and polymyxin B produced the greatest reduction in bacterial loads across all tissues examined. In the spleen, hemorrhagic necrotic lesions were nearly absent, splenic corpuscles appeared clearly defined with only mild residual inflammation, and the overall morphology closely resembled that of normal spleen tissue. In the liver, the combination group exhibited substantially reduced inflammatory infiltration, restored normal hepatic cord organization, and markedly ameliorated tissue damage, representing the most favorable outcome among all treatments. In the ileum, the combination therapy further significantly alleviated the intestinal mucosal tissue damage and promoted the recovery of villus morphology to a improved state, outperforming the protective effects observed with polymyxin B monotherapy alone.

## 4 Discussion

### 4.1 Effects of piceatannol on *E. coli* O157 biofilms formation

The data presented herein demonstrate that piceatannol possesses significant *in vitro* anti-biofilm activity against *E. coli* O157, primarily by impairing early biofilm development and compromising the structural integrity of the extracellular matrix. The marked, concentration-dependent inhibition of biofilm formation by piceatannol positions it as an effective suppressor of biofilm development. This level of inhibition is particularly noteworthy given that many phytochemicals require millimolar concentrations to exert similar effects (De Rossi et al., 2025), suggesting a high specificity or potency of piceatannol in disrupting processes (such as initial surface attachment) that are prerequisite for mature biofilm architecture (Kostakioti et al., 2013). Crucially, this anti-biofilm effect occurs at concentrations below the minimum inhibitory concentration (MIC > 256 μM), indicating that piceatannol suppresses biofilm development through mechanisms independent of direct bactericidal activity. Such sub-MIC anti-biofilm effects are increasingly recognized as desirable for combating antimicrobial resistance, as they interfere with bacterial colonization and biofilm development rather than targeting viability, thereby exerting minimal selective pressure for resistance (Pecoraro et al., 2023; Hassan et al., 2023; Bougouizi et al., 2026). In contrast to its potent inhibition of biofilm formation, piceatannol exhibited no significant eradication activity against pre-formed mature biofilms (Figure 1B), with biomass retention rates remaining near 100% across all tested concentrations. The inability of piceatannol to eradicate pre-formed mature biofilms suggests that its mechanism of action is likely directed toward preventing metabolically active processes involved in extracellular polymeric substances or initial surface attachment, rather than causing reduced viability of established sessile populations. This can be attributed to two principal factors. Firstly, the extracellular polymeric substance matrix of mature biofilms serves as a formidable diffusion barrier, physically impeding the penetration of exogenous compounds into the inner strata of the community (Donlan and Costerton, 2002; Flemming and Wingender, 2010). Secondly, biofilm-resident cells frequently adopt a slow-growing or dormant phenotype, which exhibits inherent tolerance to antimicrobial agents targeting metabolic processes (Stewart, 2003). The Live/Dead staining demonstrated the concentration-dependent effect of piceatannol on biofilm viability. Morphological analysis by SEM corroborates these findings, revealing a pronounced transition from the densely packed and intact architecture of the control group to one characterized by compromised matrix integrity and increased intercellular spacing.

### 4.2 Growth and motility suppression

Complementary to its anti-biofilm activity, piceatannol significantly impaired the physiological fitness of planktonic *E. coli* O157 by suppressing both proliferative capacity and surface motility. The concentration-dependent growth inhibition shown in Figure 2A, where treated cultures never reached the same maximum density as the control, confirms that piceatannol exerts growth-inhibitory effects at sub-MIC concentrations. Corroborating these growth profiles, CFU assays (Figure 2B) demonstrated a time-dependent reduction in bacterial viability, with the 128 μM group exhibiting significantly lower counts at 12 h. These growth-inhibitory effects could partly account for the reduction in biofilm formation by limiting the pool of cells available for initial attachment. Consistently, the metabolic activity assay confirmed a concentration-dependent suppression of metabolic output. This metabolic impairment could limit the cellular resources available for biofilm development, as active metabolism is required to support the energy-dependent processes underlying surface colonization and biofilm establishment (Öztürk et al., 2023). The suppression of swarming motility highlights a key phenotypic effect associated with the anti-biofilm activity of piceatannol, as motility is intrinsically linked to the initial stages of surface colonization. Swarming is a flagella-driven, multicellular behavior that requires high energy investment (Kearns, 2010). The dose-dependent reduction in swarm diameter indicates that piceatannol impairs this multicellular behavior, although the underlying molecular interaction remains to be investigated (Sauer et al., 2022). Given that motility is essential for surface colonization and microcolony formation, these findings suggest a possible association between diminished swarming and reduced biofilm formation. By impairing surface colonization and nutrient acquisition, piceatannol could effectively attenuates the invasive potential of *E. coli* O157.

Thus, the dual impact of piceatannol on metabolic activity and swarming motility highlights a multi-phenotype effect that extends beyond simple growth suppression. Collectively, these findings indicate that piceatannol reduces *E. coli* O157 biofilm formation could through concurrent interference with multiple phenotypes, including impaired surface colonization, suppressed metabolic output, and attenuated swarming motility, although the precise molecular targets remain to be elucidated. At the foundational level, growth inhibition limits the pool of planktonic cells available for initial surface attachment, creating a quantitative disadvantage for biofilm initiation. Concurrently, metabolic suppression constrains the cellular energy and resources required to support surface colonization. Most critically, the impairment of swarming motility directly disrupts the coordinated, surface-seeking behaviors that drive initial attachment and microcolony development. While reduced planktonic proliferation and metabolic activity undoubtedly contribute to lower biofilm biomass, the biofilm phenotype induced by piceatannol may involve mechanisms beyond simple growth inhibition. Notably, swarming motility represents a multicellular behavior governed independently of bulk growth rates, and its disruption by piceatannol reflects a targeted interference with surface-adaptive processes rather than a passive consequence of slowed growth. Therefore, piceatannol appears to function predominantly as an agent that disrupts early biofilm establishment by limiting the pool of colonizing cells, metabolic capacity, and surface-seeking motility, with growth suppression serving as a reinforcing rather than primary driver.

### 4.3 Inhibitory effects of piceatannol on EPS and ECP

The dose-dependent suppression of EPS and ECP underscores the inhibitory effect of piceatannol with the structural foundation of the biofilm matrix. The approximately 75% reduction in EPS synthesis at concentrations of 128 μM and above is particularly significant, as these polysaccharides constitute the primary scaffold for *E. coli* O157 biofilm integrity (Limoli et al., 2015). This inhibition directly explains the altered biofilm architecture observed via SEM, wherein the loss of matrix cohesion leads to cellular dispersion. Parallel reductions in ECP release further highlight the impact of piceatannol on the proteinaceous components of the matrix. The proteinaceous component of the *E. coli* O157 biofilm matrix comprises curli amyloids (Chapman et al., 2002) and accessory adhesins (Sun et al., 2022), which mediate cell–cell cohesion and surface anchoring. The ~60% reduction in ECP at high doses indicates that piceatannol not only limits polysaccharide scaffolding but also compromises the protein-based adhesin network. This dual-targeting of matrix polysaccharides and proteins impairs the formation of the extracellular matrix scaffold, undermining the integrity required for community resilience. Notably, this anti-biofilm efficacy exhibits a steep dose-dependent response and inhibitory activity declined sharply at lower concentrations (e.g., ~20% inhibition at 16 μM). Importantly, this suppression occurred at a concentration with minimal impact on planktonic growth, reinforcing that piceatannol directly interferes with matrix biosynthesis rather than indirectly reducing output through decreased bacterial density. This dissociation between matrix production and cell viability further argues against a purely growth-dependent effect, as it suggests that piceatannol selectively impairs the biosynthetic output for EPS and ECP. Coupled with the qualitative dismantling of biofilm architecture observed via SEM (Section 4.1), this targeted effect points to a mechanism whereby piceatannol compromises biofilm integrity by preventing the assembly of the extracellular matrix. Collectively, our findings suggest that the anti-biofilm efficacy of piceatannol aligns with a key role in suppressing the biosynthetic output required for matrix construction. By reducing the production of both EPS and ECP, piceatannol effectively starves the biofilm of the materials required to construct a protective microenvironment, thereby sensitising the community to external stresses and subsequent antimicrobial challenge.

### 4.4 Combination effects of piceatannol with antibiotics

The combinatorial screening revealed a complex pharmacological landscape, wherein piceatannol exhibited specific interactions with conventional antibiotics, ranging from pronounced potentiation to marked antagonism. This duality underscores that the efficacy of phytochemical adjuvants is not universal but is dictated by the specific mode of action of the partner antibiotic. The most striking potentiating effect was observed with polymyxin B, where piceatannol enhanced the antibacterial effect by over 30-fold at 1/2 MIC. These findings are consistent with a speculative hypothesis wherein polymyxin B–mediated outer membrane disruption may facilitate piceatannol access to intracellular targets, potentially suppressing bacterial recovery pathways. It is important to emphasize that this mechanism remains experimentally unvalidated and is presented here as a theoretical framework to guide future research. Consistent with the potentiating effect on antibacterial activity, the combination of piceatannol and polymyxin B also markedly suppressed Shiga toxin production, decreasing levels to approximately 2 pg./mL. This reduction was significant relative to the control (*p* < 0.001) and piceatannol alone (*p* < 0.01) but not statistically superior to polymyxin B monotherapy, indicating that piceatannol does not antagonize the toxin-suppressive effect of polymyxin B. Thus, the combination potentiates reducing bacterial burden without promoting increased toxin production upon antimicrobial exposure. Conversely, the antagonism observed with cefotaxime and doxycycline is a critical finding. This antagonism may be mechanistically linked to the cytostatic, growth-static state induced by piceatannol. Cefotaxime targets Penicillin-Binding Proteins (PBPs) during active cell division, whereas doxycycline binds the 30S ribosomal subunit to inhibit protein synthesis (Kohanski et al., 2010). Consequently, piceatannol-mediated cytostasis and metabolic suppression would diminish the availability of these essential targets, thereby attenuating the bactericidal potential of both agents. More specifically, this metabolic slowdown may trigger a generalized stress response, such as the stringent response mediated by (p)ppGpp accumulation (Salzer and Wolz, 2023), which reprograms bacterial physiology to prioritize survival, downregulating the biosynthesis of PBPs and ribosomes that serve as essential antibiotic targets. Additionally, piceatannol-induced stress may activate *marA*- or *soxS*-regulated efflux networks (Oethinger et al., 1998; Stewart et al., 2015) which confer cross-resistance by reducing intracellular antibiotic concentrations. Notably, polymyxin B differs mechanistically from cefotaxime and doxycycline in that it kills cells through physicochemical outer membrane disruption (Velkov et al., 2009), a mode of action largely refractory to metabolic slowdown or efflux activity. This distinction may account for the selective antagonism observed with specific antibiotic classes, although further studies are required to confirm this hypothesis. Intriguingly, piceatannol exhibited a potentiating effect with tetracycline hydrochloride despite its structural resemblance to doxycycline, possibly reflecting differential susceptibility to efflux or stress responses. The fact that piceatannol antagonized these specific drugs while remaining neutral toward others (e.g., ciprofloxacin, gentamicin) highlights the importance of evaluating each combination individually *in vitro*, as antagonism could permit the survival of bacterial populations and undermine the utility of certain antibiotic pairings. In summary, while piceatannol demonstrates potential as an adjuvant to polymyxin B in preclinical models, this beneficial interaction does not extend to all antibiotics. Co-administration with cefotaxime, for example, is not recommended based on the current *in vitro* data, as it may permit *E. coli* O157 survival and reduce antibiotic effectiveness. It is important to note that the interaction profiles presented herein were derived from sub-inhibitory, fixed-dose viability assays. Definitive classification of these interactions necessitates future studies employing standardized metrics, such as the Fractional Inhibitory Concentration Index (FICI) and time-kill curve analyses, to fully elucidate the pharmacodynamic landscape.

### 4.5 *In vivo* efficacy of piceatannol-polymyxin B combination

To evaluate the combination activity of this *in vitro* interaction, we evaluated the impact of the piceatannol–polymyxin B combination in a murine infection model. Consistent with *in vitro* observations, the piceatannol–polymyxin B combination demonstrated effects exceeding those of monotherapy in the murine model. Polymyxin B, a cationic lipopeptide, disrupts outer membrane integrity in Gram-negative pathogens, including *E. coli* O157 (Zakharova et al., 2022), a property that may facilitate piceatannol access to intracellular targets implicated in metabolic and biosynthetic regulation. These tissue-specific improvements and survival gains are consistent with this observed efficacy, as neither agent alone achieved comparable reducing bacterial burden or pathological restoration. The pronounced reduction in hepatic CFU counts relative to polymyxin B monotherapy aligns with the established role of the liver as a persistent niche for *E. coli* O157. By accessing these protected intracellular reservoirs, piceatannol may suppress the biosynthetic pathways required for bacterial maintenance and extracellular matrix production, thereby reducing residual populations that escape the membrane disruptive effects of polymyxin B. The even more striking reduction in splenic CFU counts relative to both the model and piceatannol-alone group further supports this complementary dynamic. Beyond bacterial burden reduction, the combinatorial regimen conferred substantial benefits for host tissue repair in the murine model, most notably the restoration of ileal villous morphology toward an improved state. While polymyxin B monotherapy partially mitigated this damage, the addition of piceatannol yielded significantly greater improvements. However, because polymyxin B may trigger bacterial lysis and Shiga toxin release, the extent to which these tissue benefits derive from virulence suppression versus altered toxin exposure remains unclear. This functional complementarity between the two agents likely contributes to the improved survival outcomes, with the combination raising 7-day survival to 80% compared to 60% with polymyxin B alone. It should be noted that these histological observations were not accompanied by formal blinded quantitative pathological scoring, which represents an important limitation of the current study. Nevertheless, the precise contribution of piceatannol to tissue regeneration versus bacterial burden reduction remains to be fully decoupled, as the complex interplay between bacterial virulence, host immunity, and phytochemical bioactivity was not dissected at the molecular level in this study.

In summary, these findings demonstrate that piceatannol enhances polymyxin B efficacy at sub-MIC concentrations and reduces the polymyxin B dose required for bacterial clearance in a murine infection model. Although validated in a murine model, the lack of direct measurement of Shiga toxin release and stx expression limits the interpretation of bacterial clearance outcomes. Further investigation is warranted to quantify toxin dynamics during combination treatment, delineate molecular targets of piceatannol, and assess the potential for resistance development. Collectively, these findings support the activity of the piceatannol–polymyxin B combination in reducing bacterial burden and mitigating tissue injury in a murine model of *E. coli* O157 infection.

## 5 Conclusion

This study characterizes piceatannol as an effective modulator of *E. coli* O157 biofilm formation *in vitro*, which effectively suppresses initial biofilm development, concurrent with suppressed swarming motility and reduced production of key matrix components (EPS and ECP). Fluorescence and microscopic observations reveal that piceatannol compromises biofilm integrity and alters cellular aggregation. These changes may limit the establishment of persistent infection. Pharmacologically, piceatannol exhibits complex interactions with conventional antibiotics. Notably, it enhances polymyxin B activity, whereas it displays antagonism with cefotaxime and doxycycline. Furthermore, the *in vivo* efficacy of the piceatannol-polymyxin B combination was demonstrated in a murine model, showing significantly reduced bacterial burden in visceral organs, improved ileal mucosal morphology, and improved survival rates compared to monotherapy. Collectively, these findings suggest that piceatannol is a potential adjuvant to enhance polymyxin B efficacy in preclinical models. This combination warrants further investigation to validate these pharmacodynamic interactions.

## Data Availability

The raw data supporting the conclusions of this article will be made available by the authors, without undue reservation.
